# Kidney transplant dysfunction in a patient with COVID − 19 infection: role of concurrent Sars-Cov 2 nephropathy, chronic rejection and vitamin C-mediated hyperoxalosis: case report

**DOI:** 10.1186/s12882-021-02298-x

**Published:** 2021-03-15

**Authors:** Urmila Anandh, Swarnalata Gowrishankar, Alok Sharma, Alan Salama, Indranil Dasgupta

**Affiliations:** 1 Department of Nephrology, Yashoda Hospitals, Secunderabad, 500003 India; 2 Department of Pathology, Apollo Hospitals, Jubilee Hills, Hyderabad, 500044 India; 3 Dr LalPathlabs National Reference Laboratory, Rohini, New Delhi, 110085 India; 4 Department of Renal Medicine, Royal Free Hospital, University College London, NW3 2PF, London, UK; 5 Department of Renal Medicine, Heartlands Hospitals, Birmingham, Warwick Medical School University of Warwick, Warwick, UK

**Keywords:** COVID-19 nephropathy, Kidney transplant, Oxalate nephropathy, Tubulo-reticular inclusions

## Abstract

**Background:**

COVID-19 infection in kidney transplant recipients often lead to allograft dysfunction. The allograft injury has various histopathological manifestations. Our case illustrates the unusual combination of allograft rejection, acute kidney injury secondary to oxalate nephropathy and SARS CoV-2 nephropathy as the cause of irreversible allograft failure.

**Case presentation:**

A 56 year old renal allograft recipient presented with a history of fever and diarrhoea for the preceding 4 weeks, tested positive for Sars-CoV2 on nasal swab and was found to have severe allograft dysfunction, necessitating haemodialysis. He subsequently underwent an allograft biopsy, which demonstrated antibody mediated rejection along with the presence of extensive oxalate deposition in the tubules. Ultrastructural examination demonstrated spherical spiked particles in the glomerular capillary endothelium and the presence of tubulo-reticular inclusions suggestive of an active COVID-19 infection within the kidney. The intra-tubular oxalate deposition was considered to be the result of high dose, supplemental Vitamin C used as an immune booster in many patients with COVID − 19 infection in India.

**Conclusions:**

This case highlights the complex pathology that may be seen in following COVID-19 disease and the need for kidney biopsies in these patients to better understand the aetiology of disease.

## Background

COVID-19 infection in kidney transplant recipients often results in serious consequences. Many require hospitalisation and have high rates of mortality [1]. Transplant recipients are at an increased risk of adverse outcomes because of their associated comorbidities and immunosuppressive therapy. A common manifestation in these patients is worsening allograft function [2]. The treatment of these patients is often challenging and need individualised care [3]. The complex therapeutic regimens along with inadequate knowledge about the mechanisms of allograft injury secondary to COVID-19 infection add to the problem in many instances as illustrated in our case.

## Case presentation

The patient was a 56 year old renal allograft recipient whose kidney was donated by his wife, 8 years previously, and was maintained on triple immunosuppression (tacrolimus, mycophenolate sodium and prednisolone). He had a past history of ischaemic heart disease, and had undergone a percutaneous transluminal coronary angioplasty two years earlier. He presented to his local hospital with a history of fever and diarrhoea, and was noted to have acute allograft dysfunction (serum creatinine on admission 3.8 mg/dl, previous reading three months earlier had been 1.2 mg/dl). Stool routine and culture as well as ultrasound of the abdomen were performed and were non-contributory. The possibility of COVID − 19 infection was considered. He was managed conservatively with intravenous fluids and antibiotics (amoxycillin sulbactam 1.2 g twice a day and metronidazole 500 mg three times a day for 7 days) to treat his diarrhoea. His mycophenolate sodium was stopped and tacrolimus dose was halved. Prednisolone was increased to 20 mg/day. Since, at the time of the first pandemic wave, early data suggested a potential benefit of hydroxychloroquine, he was started on this as well as zinc and vitamin C. However, he showed no clinical improvement, and was transferred to our hospital.

**Fig. 1:**
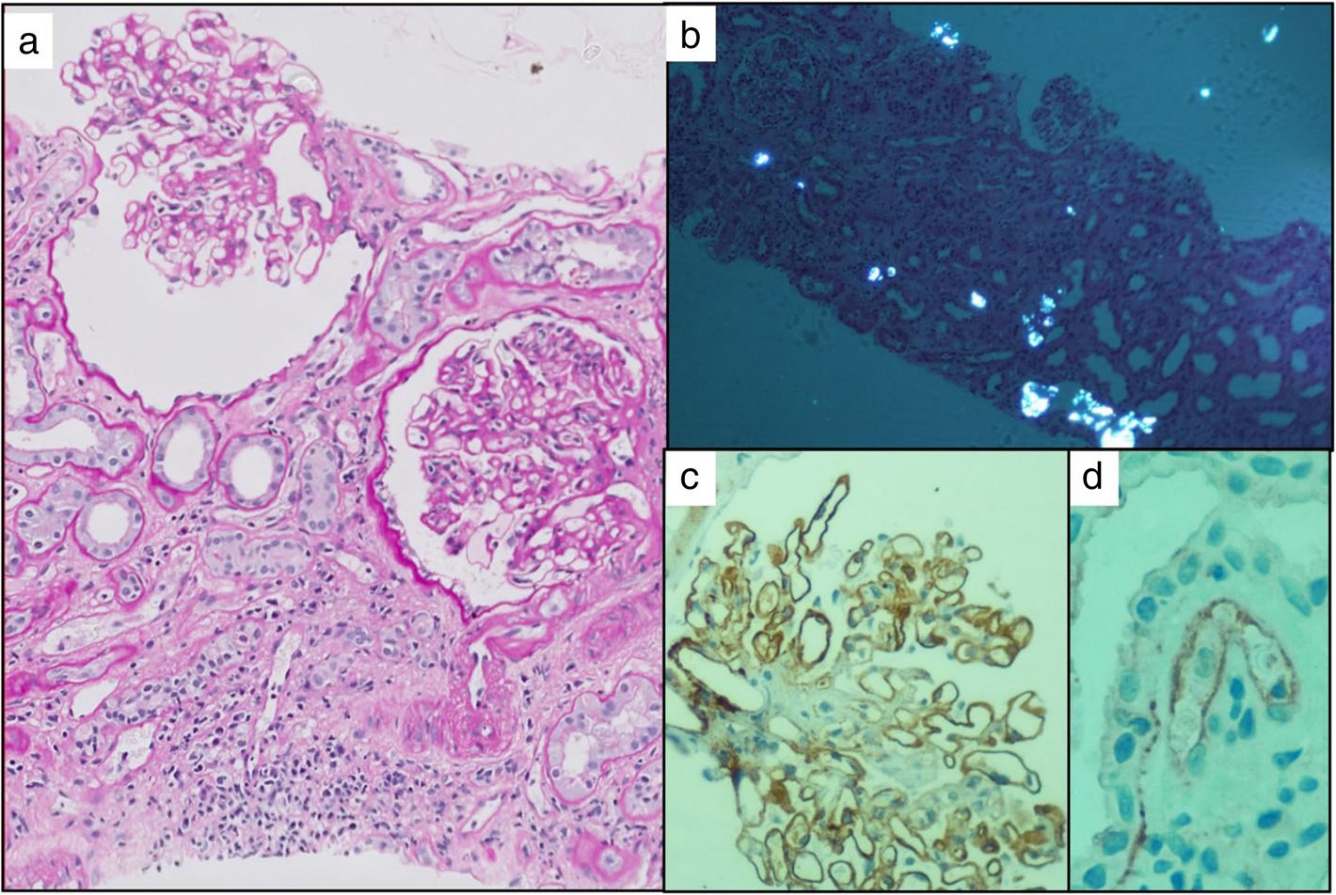
**a** Left panel shows two glomeruli with glomerulitis. A segmental duplication of the basement membrane is seen in the right one. There in mild tubular atrophy and peritubular capillaritis (PAS X400). **b** Top right panel shows the tubules viewed under polarised light having birefringent oxalate crystals within their lumina (HE X200). **c** Bottom right panel shows the C4d immune stain showing strong peripheral linear positivity along the glomerular basement membrane **d** and weak linear circumferential positivity alone one peritubular capillary (C4d immune stain -X400).(D)

In addition to his previous history, he admitted to reduced urine output, swelling of his legs and progressive breathlessness. On clinical examination, there were presence of fever, tachypnea and a low oxygen saturations of 92% on room air. Further evaluation revealed moderate left ventricular (LV) dysfunction on echocardiography associated with raised serum pro-BNP level. He was investigated for COVID − 19 infection and his SARS CoV-2 RT-PCR was positive. His serum C-reactive protein and D-dimer levels were high, and he had progressive allograft dysfunction. The 12 h trough tacrolimus levels were 5.2 ng/ml. His CT chest showed mosaic attenuation of both lungs and ground glass opacities. (Table 1) His CMV PCR showed undetectable viral counts. He was initiated on haemodialysis and treated according to our protocol for COVID-19 infection (intravenous dexamethasone, anticoagulation, remdesivir, vitamin C and antibiotics). Hydroxychloroquine was stopped. He underwent an allograft biopsy as his renal function did not improve over the following two weeks. The renal biopsy showed the evidence of glomerulitis and segmental basement membrane duplication. The tubules showed marked injury with denudation of the lining and about 20% of the tubules had intraluminal refractile oxalate crystals. There was peritubular capillary dilatation with mild to moderate capillaritis. Focal arteriolar hyalinosis and mild intimal fibrosis were also evident (Fig. 1).

**Table 1:** Laboratory and radiological investigations in our hospital

Parameter	Value	Normal Range
Haemoglobin	10.3	13–17 g%
Total Leucocyte count	4220	4000–10,000 cells/cumm
Creatinine	5.5	0.5–1.04 mg/dl
Electrolytes		
Sodium	138	137–145 mmol/L
Potassium	5.4	3.5–5.1 mmol/L
Bicarbonate	16	17–28 mmol/L
Sars Cov-2 RT PCR	Positive	
LDH	250	120–246 U/L
C-Reactive Protein	15	< 0.5 mg/dl
D-dimer	1002.1	< 500 ng/ml (FEU)
Ferritin	139	11.1–264 ng/ml
Troponin-I	0.22	0.0–0.12 ng/dl
NT Pro BNP	17,875	< 125 pg/ml
Ultrasound Abdomen	Mild Hepatomegaly Ascites Allograft doppler Resistivity Indices −0.8-.91	
Chest X Ray	Cardiomegaly Bilateral Pleural Effusions	
Computed Tomography Chest	Cardiomegaly Bilateral Pleural Effusions Mosaic attenuation of both lungs Ground glass opacities	
Echocardiography	Dilated Chambers Ejection Fraction-35% Moderate TR/MR Apex Akinetic Anterior wall Hypokinetic Minimal Pericardial Effusion

**Fig. 2:**
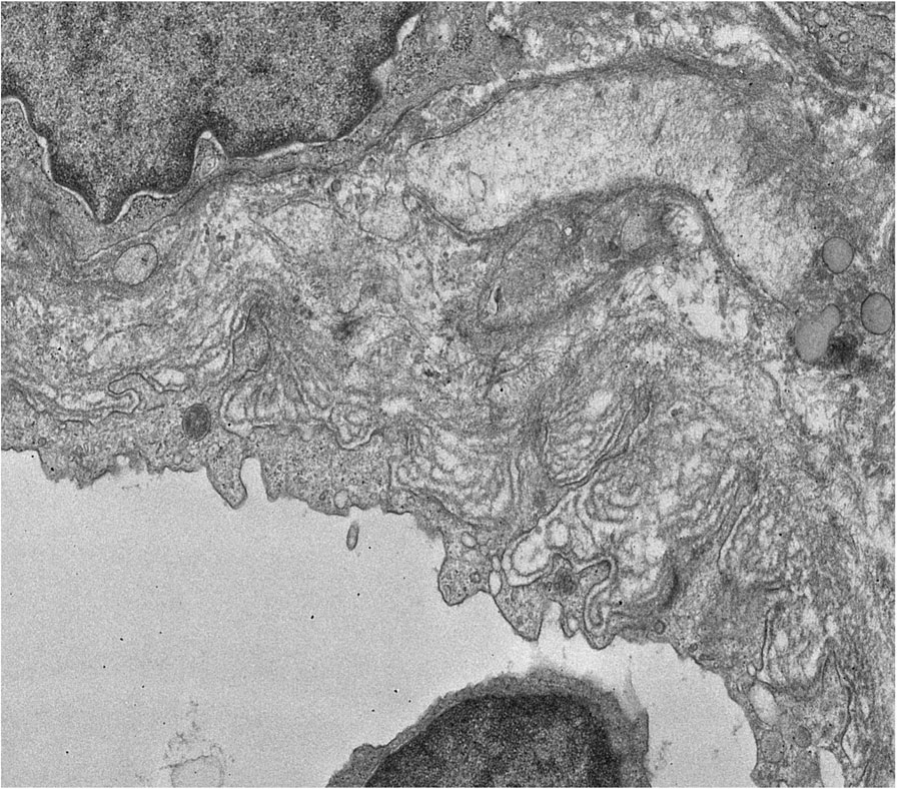
Multilayering of peritubular capillary basement membranes (TEM X 4000)

**Fig. 3:**
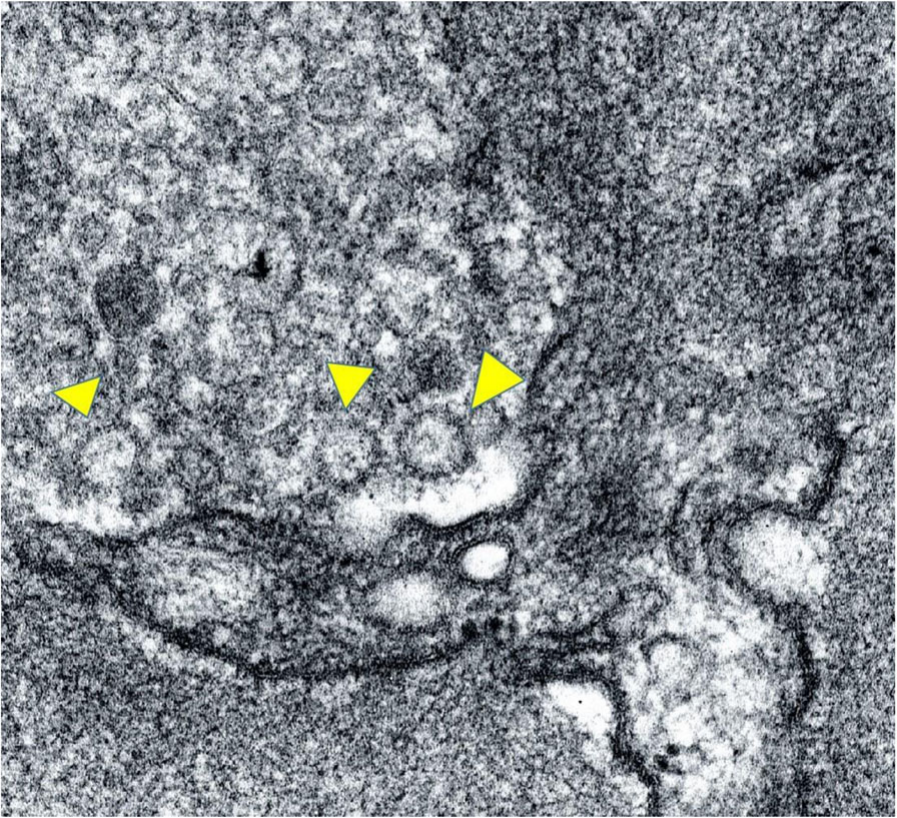
Viral particles (arrows) in glomerular endothelial cytoplasm showing peripheral “spikes” (TEM X30000)

**Fig. 4:**
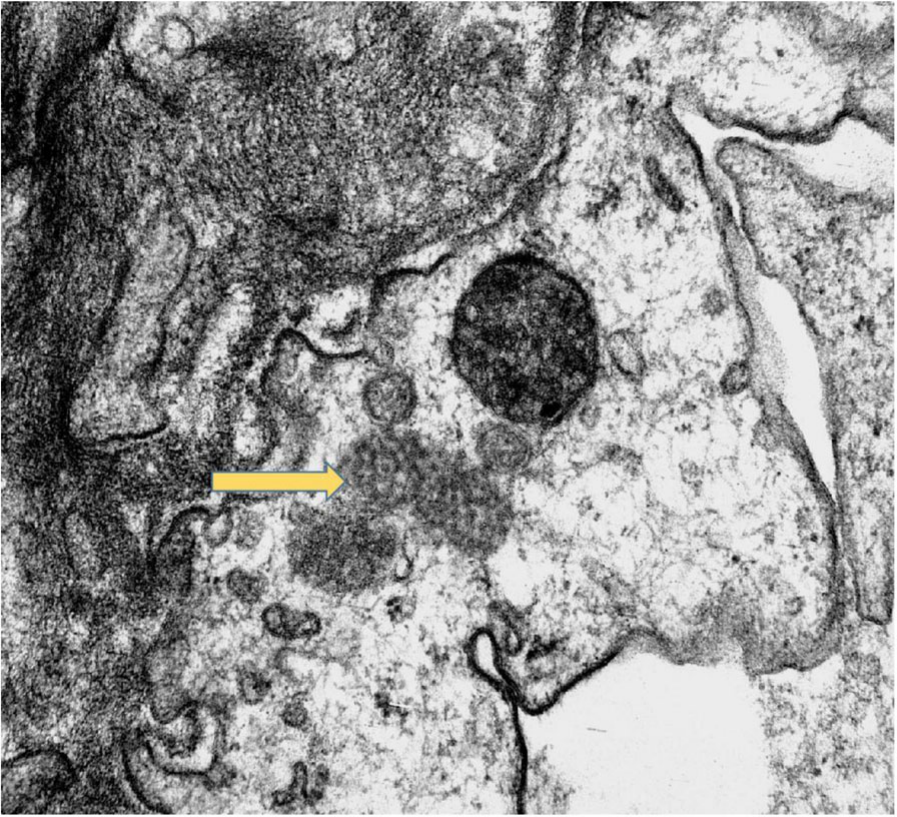
Tubulo-reticular inclusions (arrow) in glomerular endothelial cell cytoplasm (TEM X15000)

C4d immunohistochemistry showed strong peripheral linear staining along some segments of the glomerulus. Occasional peritubular capillaries (1%) also showed circumferential linear positivity (Fig. 1). The final diagnosis based on histopathology was chronic active antibody mediated rejection. There was associated acute tubular injury with extensive oxalate crystal deposition. Electron microscopy (EM) revealed prominent subendothelial rarefaction, reduplication of basement membrane and peritubular capillary basement membrane multi-layering (Fig. 2). The ultrastructural findings of spherical spiked particles (Fig. 3) and tubulo-reticular inclusions in the glomerular capillary endothelial cytoplasm (Fig. 4) were suggestive of an active COVID − 19 infection of the kidney.

**Fig. 5:**
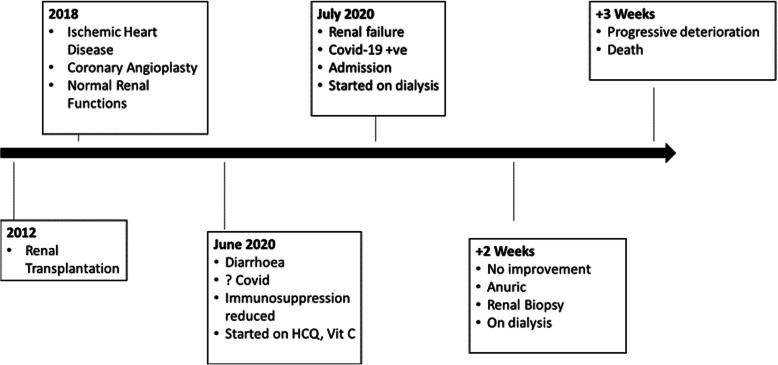
Timeline of the clinical events of the patients with emphasis on his current admission

The management of chronic active rejection requiring augmented immunosuppression was discussed with the patient and family; however, this was declined and the patient continued on intermittent haemodialysis. Respecting the family’s wish, no further investigations for the evaluation of antibody mediated rejection (such as donor specific antibody levels) and oxalate nephropathy (blood/urine oxalate levels) were done. A week after his allograft biopsy, he developed rapid deterioration of his cardiac function (repeat echocardiography showed an ejection fraction of 20%) and died. The clinical history from the time of his transplantation and his unfortunate demise is summarised as a timeline graphic. (Fig. 5).

## Discussion and conclusions

This case demonstrates a myriad of renal abnormalities related to alloimmune injury, COVID- 19 infection and its treatment. Chronic active antibody mediated rejection was confirmed by the findings of glomerulitis, early transplant glomerulopathy and mild peritubular capillaritis which was further substantiated on EM. C4d staining showed a strong linear positivity along the glomerular basement membrane (GBM) and along a few peritubular capillary walls, which was again seen as electron dense lines along the GBM on EM. Hyperinflammatory response in multiple organ systems, including renal, is believed to be common in COVID-19 infection [4], but the link between this exaggerated immune response and the risk of rejection in allograft recipients infected by the SARs CoV-2 virus is unclear [5]. Serial measurements of donor specific antibodies might have helped us in planning his immunosuppressive therapy.

The second interesting finding was that of acute tubular injury with birefringent oxalate crystals in the tubule lumina. Besides oxalate, birefringent intratubular crystal formation is seen with drugs such as cefitriaxone [6] and the proteosome inhibitor darunavir [7], but, to our knowledge, has not been reported with remdesivir. The crystals were typically fan-shaped, colourless and birefringent, and thus most likely to be oxalate crystals. This was considered to be a form of secondary hyperoxaluria as the patient did not have any previous history of stone disease. We postulate that it was related to the excessive use of vitamin C in our patient; the patient had received vitamin C at a dose of 500 mg three times a day for over a month. Though the lack of oxalate levels in blood and urine are a limitation that needs to be considered. High doses of vitamin C supplementation is commonly used in COVID- 19 infection in India to counter the potential cytokine storm. There have been three other recent reports of dialysis dependent acute kidney injury with oxalate nephropathy in patients with COVID-19; two due to excessive vitamin C intake, both recovering after more than 45 days of hospitalisation [8], and one in a patient with collapsing focal segmental glomerulosclerosis [9].

The third interesting biopsy finding was the demonstration of virus-like particles with spikes and tubulo-reticular inclusions indicative of viral infection within the endothelial cell cytoplasm. Su et al. in their autopsy series, described 9 cases with significant proximal tubular injury in whom ultrastructural studies demonstrated virus like particles in podocytes, proximal tubular epithelium and endothelium [10]. Viral-like particles were also noted in some cases in a recent biopsy series from the Mayo Clinic, but no definitive SARS-CoV-2 virions were seen in glomerular or tubular cells on electron microscopy [11]. A recent post-mortem series has been able to culture the virus thus suggesting that the virus is viable in the kidney. The authors suggest that direct viral infection may increase the risk of acute kidney injury [12]. The significance of the presence of viral particles in the glomerular endothelium and its impact on the irreversible acute kidney injury in our patient needs to be elucidated further.

Reports of COVID-19 infection in renal transplant recipients have been varied with the first report of 15 renal transplant recipients from the Columbia Transplant Registry Program reporting outcomes similar to that in the general population [2]. In contrast, a study from UK reported that 5 out of 7 transplant recipients with COVID- 19 had severe infection requiring intensive care and 1 out of 7 died [13]. The differences in outcome from centres may reflect the differences in severity of COVID- 19 infection, the treatment strategies adopted and the duration of follow up [14, 15].

Complex interactions occur when there is COVID- 19 infection in kidney transplant recipients as exemplified by this patient. Chronic active antibody mediated rejection would have been managed routinely, but the renal tubular injury was accelerated by oxalate crystals related to iatrogenic hypervitaminosis C and active COVID-19 infection of the allograft. Viral and iatrogenic injuries need to be kept in mind when reporting on these allograft biopsies and managing such cases. Physicians also should limit the use of drugs which may potentiate kidney damage in renal allograft recipients.

## Data Availability

The datasets used during the writing of the case report is available from the corresponding author on reasonable request.

## Abbreviations

COVID-19: Coronavirus disease-19
SARS CoV-2: Severe acute respiratory syndrome coronavirus 2
LDH: Lactate Dehydrogenase
RT-PCR: Real Time Polymerase Chain Reaction
NT-pro BNP: N terminal B-type Natriuretic Peptide
TR: Tricuspid Regurgitation
MR: Mitral Regurgitation
